# Unraveling Biomarker Signatures in Triple-Negative Breast Cancer: A Systematic Review for Targeted Approaches

**DOI:** 10.3390/ijms25052559

**Published:** 2024-02-22

**Authors:** Paola Pastena, Hiran Perera, Alessandro Martinino, William Kartsonis, Francesco Giovinazzo

**Affiliations:** 1 Department of Medicine, Stony Brook University, Stony Brook, Brookhaven, NY 11794, USA; 2 Renaissance School of Medicine at Stony Brook University, Stony Brook, Brookhaven, NY 11794, USA; 3 Department of Surgery, Duke University, Durham, NC 27710, USA; 4 Department of Surgery, Saint Camillus Hospital, 31100 Treviso, Italy; 5 Department of Surgery, UniCamillus-Saint Camillus International University of Health Sciences, 00131 Rome, Italy; 6 Department of Surgery, Fondazione Policlinico Universitario Agostino Gemelli IRCCS, 00168 Rome, Italy

**Keywords:** triple-negative breast cancer, biomarkers, targeted therapy, personalized medicine

## Abstract

Triple-negative breast cancer (TNBC) is one of the most aggressive subtypes of breast cancer, marked by poor outcomes and dismal prognosis. Due to the absence of targetable receptors, chemotherapy still represents the main therapeutic option. Therefore, current research is now focusing on understanding the specific molecular pathways implicated in TNBC, in order to identify novel biomarker signatures and develop targeted therapies able to improve its clinical management. With the aim of identifying novel molecular features characterizing TNBC, elucidating the mechanisms by which these molecular biomarkers are implicated in the tumor development and progression, and assessing the impact on cancerous cells following their inhibition or modulation, we conducted a literature search from the earliest works to December 2023 on PubMed, Scopus, and Web Of Science. A total of 146 studies were selected. The results obtained demonstrated that TNBC is characterized by a heterogeneous molecular profile. Several biomarkers have proven not only to be characteristic of TNBC but also to serve as potential effective therapeutic targets, holding the promise of a new era of personalized treatments able to improve its prognosis. The pre-clinical findings that have emerged from our systematic review set the stage for further investigation in forthcoming clinical trials.

## 1. Introduction

Breast cancer (BC) is the most common type of neoplasm among women in the United States, being responsible for 30% of newly diagnosed cases and ranking as the second leading cause of cancer-related deaths in more than 40,000 women annually [1]. It exhibits a significant heterogeneity in both histological and molecular aspects, resulting in different responses to therapies and overall survival. Based on gene expression profiling, breast carcinoma have been categorized into four distinct molecular subtypes: luminal A, luminal B, Triple Negative, and HER2-positive [2,3].

Triple-negative breast cancer (TNBC) accounts for 15–20% of all breast cancer and, of the several subtypes of BC, it is the most aggressive one, marked by high metastatic potential, early recurrence and a dismal prognosis. It is associated with a lower relative survival compared to the other subtypes of BC (77% versus 93%) and it contributes to 25% of all breast-cancer related mortalities [4].

The poorer outcomes compared to the other BC subtypes stem from the lack of therapeutic markers, restricting the application of targeted therapies [5]. Indeed, TNBC is characterized by the absence of estrogen and progesterone receptors (ER/PR) and absence of human epidermal growth factor receptor 2 (HER2) overexpression, making it unresponsive to the conventional therapies used for the other BC subtypes. Thus, traditional chemotherapy and radiation therapy remain the main therapeutic options for this type of neoplasm [6,7]. However, while exhibiting an elevated initial rate of response, many patients experience tumor relapse within the first three years after diagnosis, ultimately leading to a reduced disease-free survival, a phenomenon known as “Triple-negative paradox” [7,8]. 

Given the need for better therapeutic approaches, significant effort has been made over the last decade to further characterize TNBC from a molecular standpoint in order to identify signatures that could be targeted by directed therapies [9]. Indeed, while initial studies defined TNBC as a distinct molecular entity, recent evidence demonstrated this tumor to be characterized by a very heterogeneous molecular profile, and further classified it into smaller categories, including mesenchymal/mesenchymal stem cell–like (M/MSL), luminal androgen receptor type (LAR), and basal-like (BL) [10]. However, despite the numerous studies contributing to characterizing subtypes within TNBC, the underlying factors driving this cancer remain elusive [11]. Therefore, substantial research is now ongoing to better understand the molecular changes responsible for the development and progression of TNBC, with the ultimate goal of identifying novel molecular biomarkers which can be effectively targeted by therapies and which could improve the prognosis of this aggressive tumor [12,13]. 

This review aims to provide a systematic overview of the studies in the literature concerning novel molecular signatures unique to TNBC compared to other BC subtypes and healthy cells, and which could, therefore, serve as potential therapeutic targets. In particular, we focus on studies investigating the impact of inhibiting or modulating these biomarkers on TNBC cells. This will shed light on the role that these biomarkers play in tumor development and progression, crucial for a better understanding of the molecular biology of this disease. Furthermore, it will provide researchers with effective preclinical validated biomarkers which could guide future clinical research and contribute to the development of targeted approaches for TNBC patients.

## 2. Materials and Methods

We conducted a systematic review following the Preferred Reporting Items for Systematic Reviews and Meta-analysis (PRISMA) statement [14]. Due to the lack of homogeneity in study populations and inconsistencies in outcomes, we did not perform a meta-analysis.

### 2.1. Literature Search

Three databases (PubMed, Scopus, and Web of Sciences) were searched from 2008 to December 2023 to retrieve articles reporting biomarkers in triple-negative breast cancer. The following search string was used: ((Triple negative breast cancer) AND (molecular heterogeneity) OR (molecular subtypes)) AND ((biomarkers) OR (predictive biomarkers)) AND ((targeted therapy) OR (personalized therapy) OR (therapeutic approaches)). 

### 2.2. Inclusion and Exclusion Criteria

Published articles were considered for inclusion in the review if they met the following criteria: studies reporting biomarkers exclusively present in TNBC compared to non-TNBC cells or healthy normal cells, or whose increased or decreased expression further helped characterize the molecular profile of TNBC; studies exploring the mechanisms through which these biomarkers are involved in development and progression of TNBC; studies investigating the impact on TNBC cells when inhibiting or modulating these biomarkers, making them suitable for future targeted therapies. Articles that were not specific to TNBC or were not aligned with the review’s objective (such as those focused solely on prognostic biomarkers or biomarkers predictive of response to therapy) were excluded. Reviews, letters, editorials, conference papers, and book chapters were also excluded.

### 2.3. Study Selection and Data Extraction

We used the Rayyan-Intelligent Systematic review platform in order to eliminate duplicate records [15]. Two authors (P.P. and H.P.) independently screened the studies based on titles and abstracts. The same authors also independently conducted a full-text review of the articles retrieved from the first screening. A third author (A.M.) discussed with the other authors any disagreements concerning the screening process. Information from the included articles, such as the paper’s title, author’s name, date of publication, and DOI were extracted and collected in a Microsoft Excel spreadsheet (version 16.82, 24021116). 

**Figure 1:**
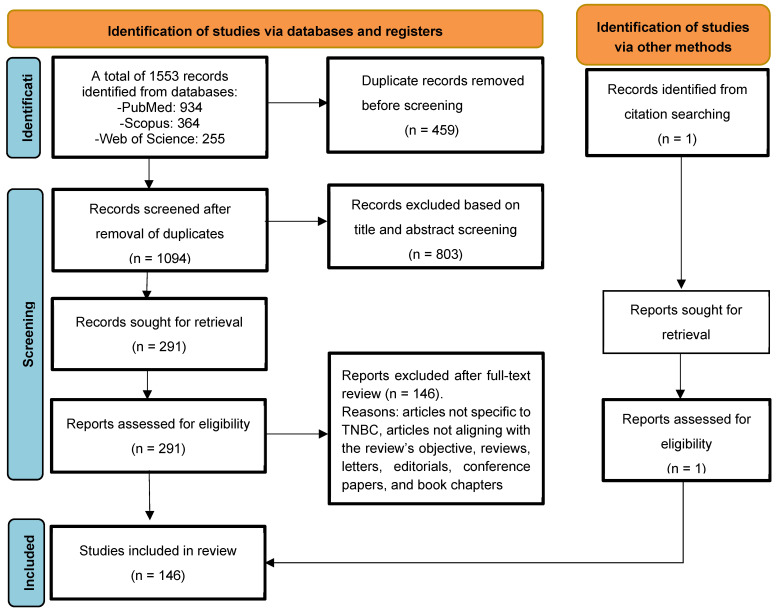
PRISMA flow diagram for selection of studies.

In Figure 1, the study flow diagram used to include the articles is reported. A total of 1553 records were identified: 934 from PubMed, 364 from Scopus, and 255 from Web of Science. After eliminating the duplicates, a total of 1094 records were screened based on the title and abstract. Of these, 803 were excluded. The remaining 291 records underwent full-text screening. 145 records were included, and 146 were excluded due to findings not specific to TNBC and not aligning with this review’s objective (those pertaining only to prognostic biomarkers or biomarkers solely predictive of treatment response). An additional record was retrieved via citation searching. Thus, a total of 146 articles published between 2008 and 2023 were included in this systematic review. 

## 3. Results

Results obtained from this systematic review have been divided into two main sections: (1) a section focusing on studies not only investigating the expression of biomarkers in TNBC, but also manipulating them with experiments in order to gain more insights into the molecular biology of the disease; (2) another section reporting biomarkers found to have a different expression in TNBC compared to non-TNBC or healthy cells, but yet to undergo targeted manipulation experiments. 

**Figure 2:**
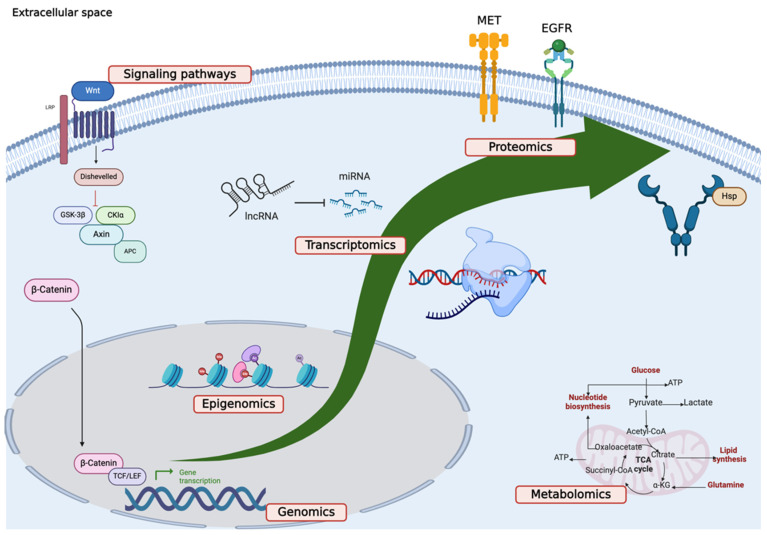
Comprehensive multi-omics analysis of TNBC.

The first section has been further subdivided into five main domains: genomics, epigenomics, transcriptomics, proteomics, and metabolomics. An additional paragraph has been dedicated to specific signaling pathways in TNBC. Figure 2 summarizes the intricate interplay of pathways responsible for TNBC development and progression. 

### 3.1. Molecular Signatures in TNBC and Impact of Their Manipulation in Pre-Clinical Studies

Numerous biomarkers have been identified as key components shaping the molecular profile of TNBC. Additionally, through rigorous experimental manipulation, they have been demonstrated to have a significant impact on the behavior of TNBC, establishing them as promising therapeutic targets in pre-clinical studies.

A total of 66 studies about novel molecular signatures in TNBC which underwent further targeted manipulation experiments have been included and reported in Table 1. 

#### 3.1.1. Genomics

Among the genes commonly found to be highly expressed in BC subtypes, increased attention has been given to kinesin family member 20A (*KIF20A*), due to increased prevalence in TNBC and its association with unfavorable prognosis. Experimental interventions aimed at inhibiting the endogenous *KIF20A* expression, either through the use of small interfering ribonucleic acids (siRNAs) or paprotrain (a selective *KIF20A* inhibitor), resulted in significant suppression of BC growth, through the arrest of the cell cycle at the G2/M phase, leading to subsequent mitotic cell death. Therefore, *KIF20A* stands as a promising biomarker that could be targeted with future therapeutic approaches in TNBC [16]. 

The Death Effector Domain-containing Protein (DEDD) has been found to be a potential vulnerability in TNBC. A pre-clinical investigation into its expression and mechanism of action revealed that over 60% of TNBC cases exhibit elevated levels of DEDD. Moreover, with its cytoplasmic localization, it plays a crucial role in promoting G1/S cell cycle transition through two mechanisms: it interacts with the heat-shock cognate 71 kDa protein (HSC70), leading to increased expression of cyclin D1, and facilitates the proteasome-mediated degradation of retinoblastoma (Rb) family proteins [17]. Due to an accelerated G1/S cell cycle progression, DEDD overexpression seemed to provide an increased susceptibility to a combination treatment involving cyclin-dependent kinases 4/6 (CDK4/6) and EGFR inhibitors, which synergistically inhibited the progression of TNBC both in vitro and in vivo. This discovery provides a compelling pre-clinical rationale for considering combinatorial regimens of CDK4/6 inhibitors for TNBC patients [17]. 

Non-SMC condensin I complex subunit D2 (NCAPD2) is a regulatory subunit participating in chromosome condensation and segregation and plays a crucial role in the cell cycle [18]. A study investigating 179 breast cancer samples revealed for the first time a connection between NCAPD2 and TNBC. Specifically, NCAPD2 emerged as a key player influencing the cell cycle and migration of TNBC cell lines, participating in the p53 signaling pathway, and its heightened expression was frequently linked to lymph node involvement. The role of NCAPD2 in TNBC was confirmed by its knockdown, which induced G2/M arrest via the p53 signaling pathway. This, in turn, led to inhibited proliferation, apoptosis, and a reduction in the invasiveness of TNBC cells. This suggests that NCAPD2 is a crucial factor in TNBC progression and represents a potential novel therapeutic target [18]. 

The Polo-like kinase (PLK) family plays an important role in regulating the cell cycle. Recent evidence showed that TNBC is characterized by a lower expression of PLK2, due to frequent loss of chromosome 5q11-35. Shedding light on PLK2′s role, experiments involving shRNA-induced knockdown resulted in a notable increase in colony numbers compared to controls. This suggests a tumor-suppressive role for PLK2, found to be mediated by a direct interaction of PLK2 with PLK1, whose overexpression is involved in TNBC growth [19]. Furthermore, in TNBC models with PLK2 deletion/low expression, treatment with PLK1 inhibitor volasertib, alone or combined with carboplatin, led to an improved treatment response, providing a rationale for using this approach to treat tumors with low or deleted PLK2 [19]. 

The phosphatase PPM1A, responsible for regulating kinase signaling pathways, has been identified as a key regulator of cell cycle progression in TNBC [20]. An analysis comparing RNA levels across breast cancer subtypes revealed that PPM1A is underexpressed in TNBC cells, and this downregulation was associated with TNBC growth. Further investigation showed that inducing the expression of PPM1A suppressed both in vitro and in vivo growth of TNBC cells, by decreasing CDK and Rb phosphorylation, inducing cell cycle inhibitors p21 and p27, and therefore blocking cell cycle progression. These findings demonstrate PPM1A’s role as a tumor suppressor, and suggest that manipulating its expression could be a viable therapeutic strategy for inhibiting TNBC growth [20].

Cyclin E, a crucial regulator of the cell cycle governing transition from G1 to S phase, plays a significant role in mediating replicative stress in cancer. Notably, cyclin E dysregulation has been reported in TNBC [21]. Interestingly, its increased expression has been associated with a higher prevalence of mutations in DNA repair genes, in particular *BRCA1/2*. The combination of a Wee1 kinase inhibitor (AZD-1775), targeting cyclin E-high tumors, with a PARP inhibitor (MK-4827), addressing DNA repair gene mutations like *BRCA1/2*, induced replicative stress and downregulated DNA repair mechanisms. This led to enhanced apoptosis both in vitro and in vivo, highlighting this combination treatment as a potential therapeutic strategy in TNBC patients harboring both alterations [21].

Friend Leukemia Virus Integration 1 (FLI-1), belonging to the E26 transformation-specific (Ets) transcription factor family, has gained recognition as an oncogene implicated in several malignancies, including breast cancer [22]. Notably, a study exploring the correlation between FLI-1 and different BC subtypes found it to be overexpressed in TNBC cells. Experiments, both in vitro and in mice, revealed that FLI-1 binds to the promoters of crucial epithelial-mesenchymal transition (EMT)-related genes (specifically *CDH1* and *VIM*) and regulates their expression, leading to EMT. Interestingly, the silencing of FLI-1 not only suppressed cancer stem cell properties in vitro but also hindered tumorigenesis in vivo, suggesting that FLI-1 could be a promising target for future therapeutic approaches in TNBC [22].

ATIP3, a mitotic-spindle associated protein encoded by the alternative splicing of the MTUS1 tumor suppressor gene, has been reported to be significantly downregulated in TNBC, correlating with high tumor grade and distant metastasis. Further experiments showed that silencing through siRNA increased tumor cell proliferation, while its restoration led to a reduction in proliferation and clonogenicity in vitro and in vivo, suggesting that ATIP3 could be a suitable biomarker and candidate for future targeted therapies [23]. 

Telomerase is a ribonucleoprotein enzyme composed of hTERT (human telomerase reverse transcriptase) and hTERC (telomeric RNA component) subunits. Its activation is a fundamental step for malignant transformation of human cells and has been found to be upregulated in TNBC cells [24]. In order to investigate the effects of telomerase inhibition on cancerous cells, Aboelela et al. targeted the hTERT gene with specific siRNAs. This approach, whether used alone or in combination with chemotherapeutic agents (specifically doxorubicin), disrupted cell survival and growth progression, and induced apoptosis of cancerous cells. In particular, in the TNBC cell lines, combination treatment led to a significant increase (*p* < 0.04) in caspase-8 activity compared to the control group within 72 h after transfection. This finding not only demonstrated telomerase inhibition to be a promising effective treatment in TNBC, but also highlighted its ability to potentiate the cytotoxic effect of chemotherapeutic agents [24]. 

ErbB-2 is part of the receptor tyrosine kinase family, which includes EGFR/ErbB-1, ErbB-2, ErbB-3, and ErbB-4. Notably, TNBC lacks its overexpression at the cytoplasmic membrane [25]. Interestingly, a study on the alternative splicing of ErbB-2 revealed that TNBC cells express both the wild type ErbB-2 and the non-canonical isoform, isoform c, within the nucleus, where they act as transcription factors binding DNA at ErbB-2 response elements (HAS). Inside the nucleus, they play a role in regulating the transcription of *Erk5*, a downstream mediator showed to be involved in the induction of TNBC growth in vivo. An inhibition of TNBC proliferation was seen after the silencing of *Erk5*, confirming its critical role in TNBC. Additionally, the eviction from the nucleus or the silencing of isoform c blocked tumor growth, highlighting the dominant oncogenic potential of isoform c and suggesting both canonical and alternative isoforms of ErbB-2 as therapeutic targets in TNBC [25]. Another study showed that the use of inhibitors of the retrograde transport Retro-2 and its cyclic derivative Retro-2.1 not only blocked NErbB-2 retrograde transport but also suppressed TNBC growth in vitro, ex vivo, and in vivo, suggesting R2 as a potential novel therapeutic agent for TNBC [26].

#### 3.1.2. Epigenomics

DNA methylation represents the most widely recognized epigenetic modification, with an important role in regulating the expression of crucial cancer-related genes. As a result, it emerged as a highly promising epigenetic biomarker for various cancers, including BC [27].

A study focused on uncovering clinically significant aberrantly methylated genes in tumor cells reported three genes to be exclusively hypomethylated in TNBC, specifically Von Willebrand factor C and Epidermal Growth Factor domain-containing protein (*VWCE*), tetraspanin-9 (*TSPAN9*), and *ADAM12*. In particular, the hypomethylation of *ADAM12* was linked to adverse outcomes. Furthermore, the silencing of *ADAM12* resulted in reduced TNBC cell proliferation and migration, suggesting that its overexpression, induced by hypomethylation, has the potential to promote TNBC cell aggressiveness and, therefore, it could represent a suitable candidate for targeted therapy in TNBC [27]. 

Protein arginine methyltransferase 5 (PRMT5) is an enzyme catalyzing methylation in histone and non-histone proteins, and plays a pivotal role in regulating oncogenic and apoptotic signals. Therefore, it has recently emerged as a potential target for cancer therapy [28]. A study by Vinet et al. demonstrated that PRMT5 exhibits a distinctive subcellular distribution in TNBC compared to healthy tissue and other BC subtypes. Additionally, the inhibition of PRMT5 by the small-molecular inhibitor EPZ015666 was shown to induce apoptosis, regulate cell cycle progression, and reduce mammosphere formation both in vitro and in vivo, with its administration significantly impeding tumor progression in a patient-derived xenograft model. Furthermore, combining EPZ015666 with the EGFR inhibitor Erlotinib showed additional benefits, suggesting PRMT5 targeting, alone or in combination, as a promising treatment strategy for TNBC [28].

Hypermethylated in Cancer 1 (*HIC1*) is a tumor suppressor gene found to be subjected to epigenetic silencing in several cancer types, including prostate, liver, colorectal, lung, and breast cancer [29]. In a pre-clinical study analyzing different breast cancer subtypes, it was observed that *HIC1* expression is silenced only in TNBC, in contrast to the other BC subtypes. The restoration of *HIC1* expression in vitro and in vivo experiments on TNBC cells significantly reduced cell migration, invasion, and metastasis. Moreover, the protein lipocalin-2 (LCN2) was found to be the critical downstream target of *HIC1*, and its autocrine secretion, induced by the loss of *HIC1*, activated the *AKT* pathway associated with TNBC progression. The HIC1-LCN2 axis emerges, therefore, as a promising target for TNBC therapy [29]. 

The overexpression of the transcription factor HOXB7 is frequently seen in BC, particularly in aggressive subtypes like TNBC. Despite its association with poor prognosis, the specific targets affected by HOXB7 are not well understood [30]. The silencing of HOXB7 in a pre-clinical study investigating the impact of HOXB7 deregulation in TNBC cells demonstrated a reduction in migration and invasion rates. Furthermore, the silenced cells exhibited increased expression of CDH1 and decreased expression of DNMT3B, along with reduced promoter methylation of CDH1. This suggests that HOXB7 influences TNBC cells by regulating the epigenetics of CDH1, potentially through the indirect upregulation of DNMT3B, which controls DNA methylation of the CHD1 promoter. Targeting HOXB7 regulation, therefore, could be a promising therapeutic approach for TNBC treatment [30].

#### 3.1.3. Transcriptomics

RNA helicase eIF4A has a pivotal role in the initiation of translation, with multiple tumor-promoting genes requiring its enhanced activity for effective protein synthesis. The vulnerability of eIF4A in TNBC has been demonstrated in a pre-clinical study by assessing the impact of eIF4A inhibition on tumor cells and the tumor immune microenvironment. Specifically, the use of the eIF4A inhibitor Zotatifin in TNBC mouse models suppressed the translation of *Sox4* and *Fgfr1*, resulting not only in the inhibition of cell proliferation but also in increased interferon response and remodeling of the tumor immune microenvironment. Additionally, Zotatifin resulted in a T cell-dependent tumor suppression by synergizing with carboplatin, suggesting Zotatifin’s potential as a part of combination therapies involving chemotherapy or immunotherapy in TNBC [31]. 

Cleavage and Polyadenylation Factor-6 (CPSF6) is an RNA binding protein involved in the alternative cleavage and polyadenylation process, facilitating RNA looping and allowing mRNA 3′ end processing [32]. Recent evidence reported TNBC to be dependent on CPSF6 for viability and tumorigenesis, making it a suitable candidate for targeted therapy. Specifically, it has been identified as a key component of the A-to-I RNA editing machinery, interacting with paraspeckles and ADAR1, and being associated with increased aggressiveness. Moreover, the utilization of prolactin (PRL) hormone, a key component in mammary differentiation pathways, has been shown to suppress this oncogenic pathway, shedding light on the significant role of differentiation pathways in tumor suppression [32]. 

Long noncoding RNAs (lncRNAs), RNA molecules exceeding 200 nucleotides without encoding structural proteins, play a pivotal role in modulating signaling pathways by interacting with proteins, DNA, and RNA, and their association with cancer initiation and progression has been well reported [33]. In particular, the lncRNA thymopoietin antisense transcript 1 (TMPO-AS1) has been found to be highly expressed in TNBC and it has been shown to impact gene signatures associated with both transforming growth factor-β (TGF β) signaling and proliferative E2F signaling pathways. Additionally, the knockdown of TMPO-AS1 hindered the proliferation and migration of TNBC cells and induced apoptosis. Its targeting with siRNA treatment suppressed the in vivo growth of primary and metastatic TNBC xenograft tumors, underscoring the key role of TMPO-AS1 in TNBC pathophysiology, positioning it as a potential therapeutic target [33]. 

Circular RNAs (circRNAs) have been associated with several diseases, with a notable impact in cancer. Their functional roles involve acting as “sponges” for microRNAs or proteins, or serving as protein scaffolds. Recent evidence showed that circRNAs play a contributory role in promoting proliferation or dissemination in TNBC [34] Unexpectedly, circular HER2 RNA (circ-HER2), encoding a novel protein HER2-103, was found to be expressed in approximately 30% of TNBC samples, and its expression was associated with a worse prognosis. The knockdown of circ-HER2 inhibited TNBC cell proliferation and invasion both in vitro and in vivo, highlighting its role in tumorigenesis. Mechanistically, HER2-103 induced the homo/hetero dimerization of epidermal growth factor receptor (EGFR)/HER3 and maintained AKT phosphorylation, thereby fostering the expression of downstream malignant phenotypes. Additionally, the use of the HER2 antibody Pertuzumab significantly reduced the tumorigenicity of circ-HER2/HER2-103 expressing TNBC cells, highlighting its potential as a therapeutic target [34].

In another investigation, circ_0000977 emerged as the most dysregulated circRNA in TNBC compared to non-TNBC. Notably, the reduced expression of circ_0000977 increased levels of miR-135b-5p within the cytoplasm of TNBC cells, and was associated with the downregulation of four genes: *GATA3, APC, LZTSI,* and *SMAD5*. Furthermore, the knockdown of miR-135b-5p led to an upregulation of APC expression. Collectively, these findings suggest the potential relevance of the circ_0000977/miR-135b-5p/APC regulatory axis in shaping the oncotranscriptomic profile of TNBC, and this axis holds promise as a prospective target for future therapeutic interventions [3].

circCAPG is a circRNA whose expression has been reported to be markedly elevated in TNBC cells compared to normal samples [35]. Additionally, the overexpression of circCAPG was found to increase the proliferation and metastasis of TNBC cells through a novel polypeptide, CAPG-171aa, which, in turn, activated the MEKK2-MEK1/2-ERK1/2 pathway. Knocking down circCAPG had a notable inhibitory effect on the growth of TNBC samples, suggesting it as a potential therapeutic target in TNBC [35]. 

The proteasome is a complex responsible for protein degradation within cells, and its disruption has been reported to contribute to several diseases, including cancer [36]. A pre-clinical study focusing on the transcriptome of TNBC showed a substantial activation of proteasome function in TNBC tissue. Specifically, the proteasome subunit beta 5 (PSMB5), a key regulator of protein function, was found to be overexpressed in TNBC. Notably, when PSMB5 was downregulated, it led to apoptosis in TNBC cells and substantially increased their sensitivity to chemotherapeutic agents, proposing PSMB5 as a biomarker and therapeutic target for TNBC [36]. 

##### Role of microRNAs in TNBC

miRNAs are small noncoding RNAs, with an average of 22 nucleotides, responsible for regulating gene expression via post-transcriptional gene silencing. Specifically, they exert their influence by binding to the 3′ untranslated region (3′UTR) of target mRNAs, leading to either degradation or translational inhibition [10,37]. Additionally, they act as controllers of cell survival and proliferation, crucial for cancer development and progression. By functioning as either oncogenes or tumor suppressors, these miRNAs are able to regulate multiple signaling pathways and, when dysregulated, can promote cancer [10]. Moreover, a substantial portion of identified miRNAs is situated within genomic regions and fragile sites associated with cancer, enhancing their potential involvement in tumor-related processes [38]. 

Recent evidence showed that miRNAs are pivotal contributors to the pathological mechanisms of breast cancer, influencing key processes like cell proliferation, metastasis, migration, and DNA methylation [2,10]. However, the impact of most miRNAs in TNBC are not yet fully understood. Therefore, current research is now focused on identifying the key dysregulated miRNAs in TNBC in order to enhance our understanding of the disease’s molecular biology and to develop effective targeted therapies [2,10]. 

A study conducted by Adams et al. investigated the role of miR-34a in TNBC [10]. By performing qRT-PCR validation experiments on a panel of 13 breast cancer and 9 normal breast cell lines, it was demonstrated that miR-34a is selectively downregulated in TNBC cell lines, specifically within the mesenchymal subtype. Given its role as a tumor suppressor regulating gene involved in growth and survival, cancerous cells suppress it to maintain a tumorigenic state. Interestingly, the reintroduction of miR-34a in TNBC cells led to suppression of oncogenic signaling pathways involved in invasion and proliferation, ultimately causing cell death through cytostasis/senescence both in vitro and in vivo. miR-34a was also found to target the proto-oncogene c-SRC and sensitize TNBC cells to dasatinib (c-SRC inhibitor), suggesting a promising combination of miR-34a-based therapies with dasatinib to target TNBC [10]. 

MiR-211-5p is another tumor suppressor playing a crucial role in TNBC progression, and standing out as a promising candidate for therapeutic targeting [2]. A pre-clinical study analyzing TNBC tissue and matched adjacent tissue demonstrated miR-211-5p to be downregulated in TNBC, particularly in the basal-like subtype, and its expression level to be associated with overall survival. Furthermore, the overexpression of this microRNA inhibited the proliferation and metastasis of TNBC cells in mice by directly targeting a sequence within the 3′-UTR of *SETBP1*, a gene whose expression is involved in cell proliferation, migration, and metastasis. Elevating miR-211-5P levels as a post-transcriptional regulator of *SETBP1* could therefore represent a potential therapeutic approach for addressing TNBC [2]. 

MiR-21 has been found to have an oncogenic role in TNBC. A study investigating miR-21 expression by RT-qPCR in TNBC specimens found that TNBC breast tissues had a significantly higher level of miR-21 compared to normal breast tissues (*p* = 0.0247) [38]. Moreover, its expression was heterogeneous within the TNBC groups. Further results revealed that increased levels of miR-21 promoted tumor growth and inhibited cell apoptosis in vitro. Additionally, reporter assay targeting *PTEN* 3′ UTR demonstrated that miR-21 targets the 3′ UTR of pro-apoptotic *PTEN*, leading to a significant decrease in *PTEN* mRNA levels [38]. 

miR-138 has been found to be a molecular signature unique to TNBC and an oncogenic driver responsible for tumor formation, suggesting it as a potential target for oligonucleotide therapies [37]. An analysis of 544 breast cancer patients unveiled a noteworthy increase in miR-138 levels in TNBC compared to luminal tumors, HER2+ tumors, or healthy breast tissue (*p* < 0.0001). Additionally, the knockdown of miR-138 induced apoptosis in vitro and prevented tumorigenesis in vivo, suggesting a pro-survival function of this microRNA in TNBC. In particular, miR-138 drives tumorigenesis by direct silencing of the pro-apoptotic tumor suppressor candidate 2 (TUSC2) through binding to its 5′-UTR target-site [37]. 

Other potential targets to be considered for TNBC therapies are miR-101 and miR-340. In the literature, miR-101 is recognized as a suppressor that hinders the migration, metastasis, and growth of BC. In TNBC, the lower expression of miR-101 is linked to an increased aggressiveness, and administering miRNA has been shown to impede cell proliferation and migration [12]. Similarly, miR-340 is reported to be a breast cancer suppressor due to its capacity to inhibit tumor cells migration and tissue invasion. Moreover, overexpressing miR-340 in TNBC cell lines led to a significant reduction in the proliferation and migration of these cells [12]. 

An elevation in the expression of miR-877-5p was found in TNBC, in particular basal-like tumor, compared to other BC molecular subtypes, correlating with reduced patient survival rates. Employing an miRNA inhibitor targeting miR-877-5p demonstrated a substantial reduction in the viability and tumor growth of TNBC models, implying that inhibiting miR-877-5p could represent a viable therapeutic strategy for TNBC treatment [39].

Recent evidence demonstrated that miR-3613-3p is significantly reduced in TNBC cell lines. In addition, when miR-3613-3p was overexpressed, there was a marked inhibition of proliferation and migration in TNBC cells, with a G1 cell-cycle arrest through the targeting of SMAD2 and EZH2. As a result, miR-3613-3P is a cancer-suppressing miRNA functioning as an anti-cancer gene whose overexpression could be a potential and innovative strategy to suppress TNBC proliferation [40].

#### 3.1.4. Proteomics

Heat shock proteins (HSPs) are molecular chaperons activating in response to stress or elevated temperatures to prevent the denaturation or unfolding of cellular proteins. Notably, heat shock protein 90 (Hsp90), found to be highly expressed in BC, plays a vital role in sustaining cancer cell survival, and is involved in several molecular processes contributing to carcinogenesis. Therefore, inhibiting its function represents a promising avenue for therapeutic intervention [41,42]. In a study targeting TNBC xenografts with the Hsp90 inhibitor, PU-H71, tumor growth was suppressed and a significant killing of TNBC cells was induced. Additionally, Hsp90 inhibition resulted in a downregulation or inactivation of the Ras/Raf/MAPK pathway and G2M phase, degradation of Akt and Bcl-XL, and inhibition of NF-kb, ERK2, Tyk2, and PKC, both in vivo and in vitro, suggesting that the anti-proliferative effect of PU-H71 directly stems from depleting TNBC cells of these malignancy-driving proteins and that Hsp90 represents a pivotal multimodal target for future therapeutic strategies [42]. In another study, the combination of PU-H71 with DHEA, a glucose-6-phosphate dehydrogenase (G6PD) inhibitor, demonstrated a synergistic anti-tumor effect on TNBC cells by suppressing Nrf2, a key transcription factor regulating antioxidants and metabolic enzymes and leading to apoptosis, suggesting that G6PD inhibitors could be valuable additions to combination therapies targeting cellular oxidative balance [41]. 

Oh et al. investigated the impact of BPD, a small molecular Hsp90 inhibitor, on TNBC cell lines. Results revealed that BPD effectively impeded TNBC cell growth by causing cell cycle arrest at G2/M phase, and induced the degradation of several oncogenic proteins, including EGFR, Her2, Met, Akt, c-Raf, and Cdk4, leading to apoptotic cell death. Additionally, the treatment inhibited MMP9 activity, crucial for tumor metastasis, suggesting BDP as a novel class of Hsp90 inhibitors able to target TNBC [43]. 

The expression of DNAJB4 (Dnaj heat shock protein family (Hsp40) member B4), a member of the human heat shock protein family (Hps40), has been found to be notably reduced in both tissue samples and cell lines of TNBC [44]. Experimental manipulation through DNAJB4 knockdown exhibited a pro-tumorigenic effect by inhibiting TNBC cell apoptosis and promoting tumor growth, both in vitro and in vivo. Conversely, the overexpression of DNAJB4 yielded opposite outcomes. Mechanistically, DNAJB4 knockdown impeded apoptosis by suppressing the Hippo signaling pathway, while this effect was reversed upon DNAJB4 overexpression, suggesting that DNAJB4 plays a crucial role in promoting TNBC apoptosis by activating the Hippo signaling pathway and could, therefore, emerge as a therapeutic target for this tumor [44]. 

Altered levels of the protein RhoGDI-2 (Rho-GDP dissociation inhibitor 2), known for its association with metastasis and poor survival, have been found in several tumors, specifically in TNBC. In this context, the targeting of RhoGDI-2 in TNBC cells by RNA interference resulted in mitochondrial dysfunction, activation of caspase-3 and -9, and increased sensitivity to cisplatin therapy, suggesting that its inhibition may be used as a therapeutic strategy in TNBC, especially when combined with cisplatin-based chemotherapy [45]. 

Paraoxonase-2 (PON2) is an enzyme which counteracts oxidative stress by decreasing the generation of reactive oxygen species (ROS). Interestingly, this enzyme has been found to be upregulated in malignant cells and to be associated with aggressiveness and chemoresistance [46]. An in-vitro study examining BC specimens demonstrated TNBC cells to be characterized by an overexpression of this enzyme in comparison to healthy cells. Additionally, PON2 knockdown led to a notable decrease in cell proliferation compared to control (*p* = 0.039 at the 48-h timepoint and *p* = 0.022 at the 72-h timepoint), suggesting it as a potential effective therapeutic target for TNBC [46]. 

Fascin-1 (FSCN1) is an actin-bundling protein whose increased expression has been documented in several types of tumors, including breast cancer [47]. Notably, a preclinical in-vitro study revealed significantly higher FSCN1 expression in TNBC compared to non-TNBC subtypes and showed FSCN1 overexpression to be associated with TNBC cell migration and invasion, identifying it as a potential biomarker of aggressiveness. Interestingly, cell migration and invasion were promoted by the activation of MAPK, triggered by epidermal growth factor-induced FSCN1 expression. Furthermore, co-treatment with FSCN1 siRNA and gefitinb resulted in a substantial decrease in FSCN1 expression compared to individual treatments, suggesting that combined inhibition of EGFR and FSCN1 could be used as a novel therapeutic strategy for TNBC [47]. 

The PDLIM2, a protein playing a crucial role in controlling the stability of transcription factors such as NF-kB and STATs in both epithelial and hemopoietic cells, has been found to be abundant in cancer cell lines displaying an epithelial-to-mesenchymal phenotype [11]. In particular, PDLIM2 protein was detected in 60% of diagnosed TNBC tumors, compared to 20% in other BC subtypes. Additionally, high levels of PDLIM2 expression were observed to be linked to adhesion signaling and increased b-catenin activity. Furthermore, the suppression of PDLIM2 has been found to reverse the epithelial-to-mesenchymal phenotype and to inhibit tumor growth in vivo, suggesting that targeting these pathways might offer viable therapeutic strategies for TNBC [11].

GRB7 is a multidomain protein that acts as a mediator for signaling pathways by interacting with tyrosine kinases, including focal adhesion kinase (FAK) and members of the ERBB family [48]. To determine the potential involvement of GRB7 in the pathobiology of TNBC, a study conducted by Giricz et al. investigated the impact of inhibiting GRB7 in TNBC cell lines. Results showed that the inhibition of GRB7 resulted in reduction in cell motility and invasion, as well as a promotion of cell death through apoptosis, suggesting that GRB7, or pathways dependent on it, could represent relevant therapeutic targets in the context of TNBC [48]. 

Clusterin (CLU) is involved in the promotion of several downstream oncogenic pathways, playing a pivotal role in oncogenesis [49]. In this context, protein kinase D3 (PRKD3) is identified as a crucial regulator, driving TNBC growth through its association with CLU. Mechanistically, by binding to CLU and inhibiting its lysosomal distribution and degradation, PRKD3 ensures CLU protein stability. An analysis of TNBC tumor samples revealed a significant elevation and positive correlation between CLU and PRKD3 protein levels. Additionally, the silencing of CLU and PRKD3, through the CLU silencer OGX-011 and the PRKDs inhibitor CRT0066101 suppressed tumor growth both in vitro and in vivo. Therefore, the targeting of the CLU pathway, through the identification of PRKD3 as its key regulator, represents a promising strategy against TNBC [49].

A large family of growth factors, characterized by a common EGF motif, and their transmembrane receptor tyrosine kinases of the EGFR family, have gained increasing attention due to their involvement in tumor progression. An increased expression of EGFR has been reported in TNBC, with its downregulation leading to a reduction in motility, signaling, and proliferation of TNBC cells [50]. After identifying an elevated MET and EGFR expression in TNBC and their correlation with poor prognosis, Linklater et al. explored the effectiveness of inhibiting MET ((MGCD265 or crizotinib) and/or EGFR (erlotinib) inhibition against TNBC progression. Results showed that the combined inhibition suppressed tumor growth and markedly diminished the fluctuations in treatment response compared to individual therapies, suggesting this dual inhibition as a potential novel therapeutic strategy for TNBC [51]. Another pre-clinical study showed EGFR to be highly expressed in basal-like TNBC, together with PYK2 and FAK, implicated in the progression and invasion of breast cancer [52]. The simultaneous targeting of EGFR and the nonreceptor tyrosine kinase PYK2/FAK synergistically inhibited the proliferation of TNBC cells in vitro and suppressed tumor growth in a mouse xenograft model, proposing a promising and efficient therapeutic strategy for basal-like TNBC. Notably, PYK2 inhibition disrupted the upregulation of HER3 triggered by EGFR antagonists, facilitating its proteasomal degradation and overcoming HER3-associated resistance. Moreover, PYK2 knockdown induced the upregulation of NDRG1, a tumor suppressor protein, which subsequently enhanced NEDD4-HER3 binding, leading to subsequent HER3 degradation, suggesting that PYK2-NEDD4-NDRG1 circuit is a central mechanism in HER3 degradation [52]. 

Claudins (CLDNs) serve as crucial proteins in preserving cell polarity and maintaining cellular homeostasis. Moreover, their ability to recruit signaling proteins suggests a potential role in regulating cell proliferation and differentiation. Emerging evidence indicates that the expression of CLDNs can serve as a valuable indicator of biochemical and functional changes occurring in both normal and neoplastic epithelial tissues. The identification of a molecular subtype of breast cancer known as claudin-low has raised considerable interest in these proteins and their potential involvement in tumorigenesis. Notably, this subtype is characterized by specific molecular signatures associated with mammary stem cells and epithelial-to-mesenchymal transition [72,73]. Interestingly, a study investigating cell lines derived from two transgenic HER2-positive mammary tumors revealed a tendency to spontaneously lose HER2 expression over time, leading to a transition toward a more aggressive nature. This progression was characterized by the acquisition of features associated with epithelial-to-mesenchymal transition (EMT) and stemness, traits typically observed in claudin-low tumors [74]. 

#### 3.1.5. Metabolomics 

A striking feature of cancer cells is their reliance on specific metabolic pathways, and their abnormal dependence is considered a key characteristic contributing to the advancement of the disease. Indeed, metabolic deregulation is recognized as a hallmark of cancer, playing a significant role in its initiation and progression. Specifically, BC is characterized by substantial metabolic heterogeneity. Therefore, targeting these metabolic alterations represents a promising therapeutic strategy [53,54]. 

In a study employing mass-spectrometry-based metabolic profiling, the investigation of cell-associated metabolite levels in 15 TNBC cell lines and non-cancerous controls revealed that TNBC cells are metabolically distinct from non-transformed normal breast cells [53]. Specifically, the levels of cellular redox buffer glutathione were found to be reduced in TNBC cell lines compared to controls. This reduction was even more pronounced in non-basal-like TNBC. These cell lines also exhibited increased sensitivity to drugs that inhibit glutathione production. This effect was subsequently rescued by the antioxidant N-acetylcysteine, revealing a reliance on glutathione to control ROS and sustain the survival of tumor cells. Additionally, elevated levels of γ–glutamylcysteine ligase, an enzyme crucial for glutathione production, corresponded to poorer patient survival. In vitro and in vivo approaches using γ–glutamylcysteine ligase inhibitors to limit glutathione precursors effectively suppressed glutathione levels and inhibited TNBC growth, suggesting that targeting glutathione production could be a promising avenue for developing treatments for TNBC, particularly the non-basal-like subtype [53].

A total of 60% of TNBC cases exhibit abnormal lipids metabolism pathways. This observation has raised significant interest in exploring metabolic-associated proteins and oncometabolites as promising therapeutic targets [54]. In a study targeting cancerous cells with dandelion extract, an herbal medicine with antitumor effects and lipid regulatory properties, changes in glycerophospholipids and unsaturated fatty acids emerged as the most prominent metabolite alterations in TNBC. Additionally, dandelion extract reduced the expression of CHKA, resulting in PI3K/AKT pathway inhibition and its downstream targets, SREBP and FADS2. Notably, picrasinoside F and luteolin exhibited the highest binding scores with CHKA, suggesting their potential as inhibitors to regulate glycerophospholipids metabolism in TNBC [54]. 

Another study revealed that TNBC cells display decreased mitochondrial respiration and increased glycolysis compared to receptor-positive cells. This phenotype is caused by the dampening of mTOR pathway and low expression of p70S6K. Reintroducing p70S6K in TNBC cells reversed their metabolism to an active oxidative phosphorylation state. These metabolic changes offer, therefore, a biochemical foundation for the development of novel therapeutic approaches aimed at effectively eliminating TNBC cells through the inhibition of glycolysis [55].

A study analyzing glycolysis-related gene transcript levels across different breast cancer subtypes revealed a unique glycolysis gene signature, comprising *ENO1*, *SLC2A6*, *LHDA*, *PFKP*, *PGAM1*, and *GPI*, with the transcription factor Y-box binding protein 1 (YBX1) being strongly linked to this glycolytic profile [56]. Notably, YBX1 was found to be overexpressed in TNBC, positively correlating with epithelial-to-mesenchymal transition genes. The knocking down of YBX1 resulted in the suppression of these glycolytic genes, the reduced expression of EMT-related genes, and decreased tumor migration and invasion, highlighting not only the relevance of YBX1-glycolysis-EMT network in TNBC, but also suggesting its potential as a valuable metabolic target in TNBC patients [56].

Pyruvate kinase M2 (PMK2) is involved in the metabolic aspects of oncogenesis, contributing to the growth and invasion of tumor cells in breast and ovarian cancer. Notably, a significant upregulation of PKM2 has been observed in TNBC [57]. To unravel the signaling pathways influencing the PKM2 phenotype in TNBC, Apostolidi et al. conducted in vitro and in vivo experiments manipulating phosphorylated PKM2 and revealed a connection between the phosphorylation of PKM2 at S37 and the cyclin-dependent kinase (CDK) pathway in TNBC cells. Additionally, treatment with TEPP-46, the CDK inhibitor dinaciclib, or their combination reduced tumor growth and decreased PKM2pS37, suggesting the potential use of CDK inhibitors and pyruvate kinase activators, either alone or in combination, as promising therapeutic strategies for TNBC patients [57]. 

#### 3.1.6. Signaling Pathways in TNBC

In the pursuit of novel approaches to address TNBC, increasing attention has been given to signaling pathways involved in its tumorigenesis. In particular, aberrant activation of Wnt Pathway and the nuclear factor-kappa B (NF-κB) pathway seem to play a relevant role in TNBC and can, therefore, be suitable candidates for targeted therapy [9,58]. 

TNBC exhibits an overexpression of canonical Wnt signaling components. This was validated in a study analyzing the expression of experimentally induced oncogenic Wnt/β-catenin genes in TNBC cells. The direct link between pathway disturbance and metastasis-related characteristics in TNBC cells was demonstrated by disrupting the functionality of the Wnt/β-catenin pathway through the use of pharmacological Wnt antagonists (WntC59, sulindac sulfide) or SiRNA-mediated genetic manipulation targeting β-catenin [59]. Another study that conducted a microarray analysis for the investigation of pathways in TNBC compared to non-TNBC revealed that TNBC is characterized by an upregulation of several genes within the Wnt canonical signaling pathway (Wnt/b-catenin pathway), in particular the frizzled homolog 7 (*FZD7*). The subsequent knockdown of FZD7 with shRNA led to a reduction in cell proliferation, inhibition of invasiveness, and colony formation through the silencing of the canonical Wnt signaling pathway, as demonstrated by the decrease in the nuclear accumulation of β-catenin and a diminished transcriptional activity of TCF7. This suggests that the *FZD7*-involved canonical Wnt signaling pathway plays a key role in the initiation and progression of TNBC and, therefore, represents a promising therapeutic target [58]. 

Notable changes in NF-kB and Wnt/β-catenin signaling have also been reported in another investigation analyzing tumor-initiating cells in TNBC. Moreover, the inhibition of NF-kB signaling in this cell population led to a significant decrease in mammosphere formation and tumor initiation, emphasizing that targeting these altered pathways could be a promising avenue for future therapeutic approaches [9]. 

### 3.2. Untargeted Molecular Signatures in TNBC

Several other biomarkers have been found to have a different expression in TNBC compared to non-TNBC or healthy cells, however they haven’t undergone targeted manipulation experiments to validate their significance. All of these biomarkers have been linked to worse outcomes among TNBC patients (such as worse overall survival), indicating that their thorough investigation and manipulation in future studies could prove beneficial and lead to successful targeted therapies. 

Our analysis retrieved 77 studies reporting biomarkers yet to undergo targeted manipulation experiments. They are summarized in Table 2. 

Interestingly, differences in expression patterns were seen after chemotherapy. As an example, N-cadherin was overexpressed in TNBC only post-chemotherapy [75]. Other special cases include IGF-1R, PD-L1, CD40/OX40L [76,77], or CD147 being overexpressed pre- and post-chemotherapy [78].

**Table 2:** Biomarkers yet to undergo targeted manipulation experiments.

Author (Year)	Biomarkers	Biomarker Expression	Controls
Abuderman (2020) [79]	MFAP5, ITM2A	⇧, ⇩	None
Al-Saraireh (2021) [4]	CYP4Z1	⇧	Healthy cells
Al-Zahrani (2018) [80]	Sox10	⇧	Non-TNBC
Al-Twigeri (2022) [81]	AUF1	⇧	Non-TNBC
Ameh-Mensah (2021) [82]	Bcl-2, p53	⇧	Non-TNBC
Andrade (2020) [83]	miR-221, miR-1305, miR-4708, RMDN2	⇩	None
Avery-Kiejda (2014) [84]	miR-130a, miR-1280, miR-590-5p, miR-1308, miR-17	⇩	Non-TNBC
Bahnassy (2015) [85]	VEGF-A, IGF-I, IGF-IR, TGF-β1	⇧	Non-TNBC
Bao (2019) [86]	miR-455-5p, FOXC1, FAM171A1, RGMA	⇧	Non-TNBC and healthy cells
Bar (2017) [87]	miR-210	⇧	Healthy cells
Bertoli (2021) [88]	miR-135b, miR-365	⇧, ⇩	Healthy cells
Bhargava (2011) [76]	IGF-1R	⇧	Non-TNBC and healthy cells
Bogan (2017) [89]	BRCA-1 IRIS	⇧	Non-TNBC
Bouchal (2015) [90]	STMN1, TMSB10	⇧	Non-TNBC
Cabezon (2013) [91]	Mage-A4	⇧	Non-TNBC
Camorani (2018) [92]	PDGFRβ	⇧	Non-TNBC
Cheng (2012) [93]	HSP90 *	⇧	Non-TNBC
Cheng (2022) [94]	CDKN2A	⇧	Non-TNBC
Chung (2015) [95]	SIRT1	⇧	Non-TNBC
Cisneros-Villaneuva (2021) [96]	LINC00460	⇧	Non-TNBC
Daniels (2016) [97]	Methylated BRCA1	⇧	Non-TNBC
Darbeheshti (2019) [98]	FOXM1, ESR1, mir-135b, mir-29b	Differential	Non-TNBC
Darbeheshti (2021) [99]	hsa_circ_0044234	⇩	Non-TNBC
Darbeheshti (2022) [100]	miR-182-5p	⇧	Non-TNBC and healthy cells
De Palma (2020) [101]	LINC01087	⇩	Non-TNBC
Eichelser (2014) [102]	miR-373	⇧	Non-TNBC
El Ayachi (2019) [103]	WNT10B network: HMGA2 and EZH2	⇧	Non-TNBC
Elfgen (2019) [104]	PIK3CA	⇩	Non-TNBC
Fan (2019) [105]	hsa-miR-148b, hsa-miR-203a, hsa-miR-203b, hsa-miR-3922	Differential	Non-TNBC and healthy cells
Fauteux (2015) [106]	MUC16, CT83, FAP, ADAM12, LRP8	⇧	Non-TNBC and healthy cells
Figenschau (2018) [107]	ICAM1	⇧	Non-TNBC and healthy cells
Gazinska (2022) [77]	PD-L1, CD40/OX40L	⇩	Non-TNBC
Goncalves (2023) [108]	Vimentin	⇧	Non-TNBC
Guo (2016) [109]	EZH2	⇧	Non-TNBC
Herrera (2012) [110]	TNF-a, TGF-b	⇩	Non-TNBC
Ilgin (2020) [111]	Cytoplasmic CXCR1, cytoplasmic CD133, nuclear CD133	⇧	Non-TNBC and healthy cells
Jiang (2019) [112]	ERBB2, CDKN2A, PIK3CA	Differential	Non-TNBC
Jin (2020) [113]	Sox 10 expression	⇧	Non-TNBC and healthy cells
Kothari (2020) [114]	TBC1D9, MFGE8	⇩, ⇧	Non-TNBC
Kim (2016) [115]	GATA3	⇧	None
Le UQ (2022) [116]	Dear1	⇧	Non-TNBC
Li Q (2017) [117]	RGS20	⇧	Non-TNBC and healthy cells
Limsakul (2023) [118]	LYN, CSF1R, FGRF2, SRMS	Differential	None
Lin (2021) [119]	BATM; BATM + prostaglandin E2 receptor 3	⇧	Non-TNBC
Liu (2016) [120]	LncRNAs: ENST00000443397, ENST00000447908, NR_003221, TCONS_00000027	⇧	Non-TNBC
Liu (2018) [78]	CD147	⇧	Non-TNBC
Magalhães (2022) [121]	hsa_circ_0072309	⇩	Healthy cells
Mcnamara (2016) [122]	Erb *	⇩	None
Millis (2015) [123]	PIK3CA, EGFR *, AR, Ki-67, PTEN, topo-isomerase 1	Differential	Non-TNBC
Mirandola (2017) [124]	SP17	⇧	Non-TNBC and healthy cells
Nath (2024) [125]	SLC7A11	⇧	Non-TNBC
Nelson (2016) [75]	N-cadherin	⇧	TNBC pre-chemotherapy
Noonan (2018) [126]	KISS1R and fibulin-3	⇧	Non-TNBC and healthy cells
Novelli (2008) [127]	ERb	⇧	Non-TNBC
Okcu (2021) [128]	GLUT-1	⇧	Non-TNBC
Privat (2018) [129]	SOD1, MGST3, PRDX1; PFN1, ITGB1, ARGLU1, ANXA1	⇧	Non-TNBC
Purwaha (2018) [130]	sphingomyelin, ceramides, sphingoid base intermediates	Differential	None
Qattan (2017) [131]	miR-195; hsa-miR-195, let-7miRNAs	Differential	Non-TNBC and healthy cells
Qian (2020) [132]	IL-17	⇧	None
Rahman (2021) [133]	909 lncRNAs and 1901 mRNAs; TCONS_00076394, TCONS_00051377	Differential	None
Roseweir (2017) [134]	Phosphorylated AR-515	⇧	Non-TNBC
Santarpia (2021) [135]	FBXW7 mutations	⇧	Non-TNBC
Sayed (2013) [136]	PARP-1, TOPO- 2A, VEGF, C-MYC, bFGF, MMP-2	⇧	Non-TNBC and healthy cells
Suman (2016) [137]	A2M	⇧	Non-TNBC and healthy cells
Sundaram (2020) [138]	EpCAM	⇧	Non-TNBC
Thalor (2022) [139]	CT83, BCL11A, S100B and POU2AF1	⇧	Non-TNBC
Wang (2015) [140]	EGFR *, p53	⇧	Non-TNBC
Wang (2017) [141]	PIK3CA mutations, p-mTOR	⇧	None
Xi (2018) [142]	AFAP1-AS1	⇧	Non-TNBC
Xiao (2017) [143]	miR-128	⇩	Non-TNBC and healthy cells
Yehia (2015) [144]	HIF-1α	⇧	Non-TNBC
Zeindler (2019) [145]	Nectin-4	⇩	None
Zhang (2014) [146]	BIRC5, CENPA, FAM64A	⇧	Non-TNBC and healthy cells
Zhao (2023) [147]	miR-15b-5p, miR-148a-3p, miR-148a-3p	⇩	Non-TNBC
Zhong (2020) [1]	FaBP7, arT3, cT83, TTYH1	⇩	Non-TNBC
Zhu (2015) [148]	BRCA1 promoter methylation	⇧	Non-TNBC

⇩ designates underexpression; ⇧ designates overexpression; “differential” designates the biomarkers listed were a mixture of overexpressed or underexpressed; biomarkers with an asterisk designate biomarkers that were eventually tested in other studies and listed in Table 1.

## 4. Discussion

TNBC is marked by high metastatic potential, early recurrence and an unfavorable prognosis. TNBC lacks targetable receptors, leading to reliance on traditional therapies with suboptimal outcomes. The absence of key receptors limits targeted therapies, prompting research into novel biomarkers for potential interventions. 

The analysis of our systematic review reveals TNBC to display a heterogeneous molecular profile and reports promising biomarkers unique to this type of cancer, offering insights into their role in tumor development. These biomarkers hold potential for personalized treatments, raising hopes for improved prognosis. The exploration spans genomics, epigenomics, transcriptomics, proteomics, and metabolomics, shedding light on the multifaceted nature of TNBC.

The genomics section highlights several promising biomarkers for targeted therapy in triple-negative breast cancer (TNBC). Kinesin family member 20A (*KIF20A*) and Death Effector Domain-containing Protein (DEDD) show potential as therapeutic targets, with experimental interventions demonstrating a significant inhibition of TNBC growth. Combinatorial regimens involving cyclin-dependent kinases 4/6 (CDK4/6) and EGFR inhibitors prove effective in inhibiting TNBC progression. Non-SMC condensing I complex subunit D2 (NCAPD2) and Polo-like kinase (PLK) family members, including PLK2, exhibit relevance in TNBC. The phosphatase PPM1A, Cyclin E, Friend Leukemia Virus Integration 1 (FLI-1), ATIP3, Telomerase, and ErbB-2 isoforms further contribute to the landscape of TNBC biomarkers, offering potential for targeted therapeutic strategies.

The epigenomic perspective adds another layer to the understanding of TNBC, with DNA methylation alterations in genes like *ADAM12*, *PRMT5*, *HIC1*, and *HOXB7* being implicated. The hypomethylation of *ADAM12* is associated with adverse outcomes, emphasizing its potential as a therapeutic target to mitigate TNBC cell aggressiveness. The distinct subcellular distribution of *PRMT5* in TNBC and its inhibition inducing apoptosis and regulating cell cycle progression open avenues for targeted therapy, possibly in combination with EGFR inhibitors.

Building upon these genomic and epigenomic insights, the systematic review delves into transcriptomic alterations, shedding light on dysregulated RNA molecules. The RNA helicase eIF4A and the RNA binding protein CPSF6 emerge as pivotal players, their dysregulation influencing critical steps in protein synthesis. Long noncoding RNAs (lncRNAs), exemplified by TMPO-AS1, and circular RNAs (circRNAs), such as circ-HER2, add layers of complexity to TNBC’s molecular signature, demonstrating their potential as therapeutic targets. The comprehensive exploration of microRNAs (miRNAs) in triple-negative breast cancer (TNBC) offers a nuanced understanding of their pivotal role in regulating gene expression and influencing crucial processes in cancer development. MiR-34a, selectively downregulated in mesenchymal TNBC subtypes, exhibits tumor-suppressive effects by inhibiting oncogenic signaling pathways. Similarly, miR-211-5p, identified as a tumor suppressor, hinders proliferation and metastasis in TNBC cells. The upregulation of miR-21 in TNBC correlates with increased tumor growth, suggesting its candidacy for intervention. MiR-138, unique to TNBC, emerges as an oncogenic driver, advertising it as a target for oligonucleotide therapies. MiR-101 and miR-340, known breast cancer suppressors, show promise in impeding TNBC cell aggressiveness. Elevated miR-877-5p in TNBC, especially basal-like tumors, correlates with reduced viability, indicating its potential as a therapeutic target. MiR-3613-3p, validated through reduced proliferation and migration in TNBC cells, presents a compelling cancer-suppressing miRNA. The identified miRNA biomarkers, thoroughly examined and validated through various experimental approaches, provide compelling evidence of their effectiveness as potential therapeutic targets.

A diverse array of proteomic biomarkers has been rigorously examined and proven effective in influencing TNBC’s behavior. Heat shock protein 90 (Hsp90), a key player sustaining cancer cell survival, is successfully targeted by inhibitors like PU-H71 and BPD, showing significant anti-proliferative effects and inhibiting malignancy-driving pathways. The modulation of DNAJB4 highlights its pivotal role in promoting TNBC apoptosis through the activation of the Hippo signaling pathway, positioning it as a potential therapeutic target. RhoGDI-2 inhibition demonstrates enhanced sensitivity to cisplatin therapy, providing a compelling strategy for combination therapies in TNBC. The enzyme paraoxonase-2 (PON2) emerges as a promising target due to its association with TNBC aggressiveness, and its knockdown exhibits a notable decrease in cell proliferation. Fascin-1, GRB7, and Clusterin (CLU) are identified as biomarkers influencing TNBC cell migration, invasion, and growth, offering potential targets for combined inhibition strategies. The study on growth factors like EGFR in TNBC underlines their significance, and the inhibition of MET and EGFR, as well as the simultaneous targeting of PYK2/FAK, present promising therapeutic approaches for TNBC.

Metabolic aberrations, such as altered glutathione levels and dysregulated lipid metabolism, have been rigorously examined and proven effective as potential therapeutic targets. The modulation of these metabolic biomarkers not only provides insight into TNBC’s distinct metabolic profile but also offers a basis for developing novel treatments. Additionally, the dysregulation of signaling pathways, particularly the Wnt and NF-κB pathways, has been implicated in TNBC tumorigenesis. Experimental disruptions of these pathways, either through pharmacological interventions or genetic manipulation, demonstrated tangible effects on TNBC characteristics, validating their relevance as candidates for targeted therapies.

The omics studies conducted on TNBC reveal a complex molecular landscape where various layers of information deeply interconnect. The interplay between omics layers not only reinforces the multifaceted nature of TNBC but also highlights that genetic, epigenetic, transcriptional, and protein-level changes are not isolated; instead, they collectively contribute to the heterogeneity and complexity of TNBC. Genomic analysis establishes the fundamental genetic basis for TNBC pathogenesis. Epigenetic modifications, particularly DNA methylation, impact gene expression at the genomic level. MicroRNAs, acting as pivotal regulators in transcriptomics, connect with proteomics, demonstrating consistent patterns. For example, miR-21, identified in transcriptomics, correlates with decreased PTEN levels in proteomics. Proteomic biomarkers, such as Hsp90 and RhoGDI-2, shed light on the protein-level consequences of genomic and transcriptomic alterations, influencing TNBC behavior. The metabolomics perspective adds another layer, with findings on glutathione and glycolysis-related genes aligning with earlier genomic, transcriptomic, and proteomic identifications. This interconnectedness emphasizes the need for a holistic, multi-omic approach for a comprehensive multidimensional understanding of TNBC’s molecular intricacies and potential therapeutic targets.

Finally, the identification and categorization of biomarkers in TNBC reveal a complex landscape, with some biomarkers yet to undergo targeted manipulation experiments. Currently, a notable number of biomarkers remain unexplored in terms of therapeutic intervention. Intriguingly, variations like the chemotherapy-induced overexpression of N-cadherin suggest that certain biomarkers may only become targetable in the subset of TNBC cells that survive specific treatments, adding a layer of complexity to potential therapeutic strategies. Further investigations and targeted experiments are crucial to unlocking the full therapeutic potential of these biomarkers in TNBC.

## 5. Conclusions

Our findings underscore the diverse molecular landscape inherent to TNBC. The identified biomarkers not only distinguish TNBC but also present themselves as promising targets for effective therapeutic interventions. This revelation opens the door to a transformative era in healthcare, where personalized treatments tailored to the unique molecular features of TNBC offer the potential to enhance its prognosis significantly. The pre-clinical insights gleaned from our systematic review not only contribute to a better understanding of the molecular biology of this aggressive disease, but also pave the way for continued exploration in upcoming clinical trials.

## Figures and Tables

**Table 1:** Experimentally manipulated biomarkers in TNBC.

Author (Year)	Biomarker	Biomarker Expression in TNBC	Impact of Its Manipulation
Nakamura (2020) [16]	KIF20A	Increased expression	Use of KIF20A inhibitor arrested cell cycle at the G2/M phase and induced mitotic cell death.
Ni (2019) [17]	DEDD	Increased expression	DEED overexpression provides an increased susceptibility to the combined use of CDK4/6 and EGFR inhibitors. When used, the combination inhibited progression of TNBC.
Zhang (2020) [18]	NCAPD2	Increased expression	Knockdown of NCAPD2 induced G2/M arrest via p53 signaling pathway, leading to apoptosis.
Gao (2021) [19]	PLK2	Decreased expression	Knockdown increased colony numbers. Treatment with PLK1 (overexpressed in TNBC) inhibitors improved treatment response.
Mazumdar (2019) [20]	PPM1A	Decreased expression	Induction of PPM1A expression suppressed TNBC growth, by decreasing CDK and Rb phosphorylation, and inducing cell cycle inhibitors p21 and p27.
Chen (2021) [21]	Cyclin E	Increased expression (associated with increased BRCA ½ mutations)	The combination of Wee1 kinase inhibitor and PARP inhibitor induced replicative stress and downregulated DNA repair mechanisms, leading to apoptosis.
Yan (2018) [22]	FLI-1	Increased expression	Silencing of FLI-1 suppressed cancer stem properties and suppressed tumorigenesis.
Rodrigues-ferreira (2009) [23]	ATIP3	Decreased expression	Silencing of ATIP3 increased tumor cell proliferation, while ATIP3 restoration led to a reduction in proliferation and clonogenicity.
Aboelela (2020) [24]	Telomerase	Increased expression	Targeting the hTERT gene with siRNA, alone or in combination with doxorubicin, disrupted cell survival and growth progression, inducing apoptosis.
Chervo (2020) [25]	ErbB-2	Expression of wild type and non-canonical isoform c in the nucleus	Silencing of Erk5, downstream mediator of ErB-2, inhibited TNBC proliferation. Eviction from the nucleus or silencing of isoform c blocked tumor growth.
Madera (2022) [26]	ErB-2	Expression in the nucleus	Inhibitors of the retrograde transport of NErbB-2 blocked transport and suppressed TNBC growth.
Mendaza (2020) [27]	ADAM12	Increased expression (due to hypomethylation)	Silencing of ADAM12 reduced TNBC cell proliferation and migration.
Vinet (2019) [28]	PRMT5	Distinctive subcellular distribution	Inhibition of PRMT5 by the small-molecular inhibitor EPZ015666 induced apoptosis, regulated cell cycle progression, and reduced mammosphere formation. Combining EPZ015666 with EGFR inhibitor Erlotinib showed additional benefits.
Cheng (2014) [29]	HIC1 (and target LCN2)	Decreased expression	Restoration of HIC1 expression reduced cell migration, invasion, and metastasis.
Paco (2021) [30]	HOXB7	Increased expression	Silencing of HOXB7 reduced migration and invasion rates, along with increased expression of CDH1, and decreased expression of DNMT3B.
Zhao (2023) [31]	eIF4A	Increased expression	Use of eIF4A inhibitor (Zotatifin) suppressed translation of Sox4 and Fgfr1, resulting in inhibition of cell proliferation, increased interferon response, and remodeling of tumor immune microenvironment. Zotatifin in combination with carboplatin led to T-cell dependent tumor suppression.
Binothman (2017) [32]	CPSF6	Key component of the A-to-I RNA editing machinery (interacting with paraspeckles and ADAR1)	Use of prolactin, a key component of mammary differentiation, suppressed this oncogenic pathway.
Mitobe (2020) [33]	LncRNA TMPO-AS1	Increased expression	Knockdown of TMPO-AS1 inhibited proliferation and migration of TNBC cells, and induced apoptosis. Targeting with siRNAs suppressed growth of primary and metastatic TNBC.
Li (2020) [34]	Circ-HER2 (encoding HER2-103)	Increased expression	Knockdown of circ-HER2 inhibited TNBC proliferation and invasion. Use of Pertuzumab reduced tumorigenicity of circ-HER2/HER2-103.
Darbeheshti (2023) [3]	Circ_0000977	Decreased expression	Decreased expression of circ_0000977 increased levels of miR-135b-5p and downregulation of GATA3, APC, LZTSI, and SMAD5. Knockdown of miR-135b-5p led to an upregulation of APC expression.
Song (2023) [35]	CircCAPG (and CAPG-171aa)	Increased expression	Knockdown of circCAPG inhibited TNBC growth.
Wei (2018) [36]	PSMB5	Increased activation	Downregulation of PSMB5 led to apoptosis of TNBC cells and increased sensitivity to chemotherapeutic agents.
Adams (2016) [10]	miR-34a (and its target c-SRC)	Decreased expression	Reintroduction of miR-34a led to suppression of oncogenic signaling pathways involved in invasion and proliferation, causing cell death through cytostasis/senescence. miR-34a sensitized TNBC cells to dasatinib (c-SRC inhibitor).
Nama (2019) [37]	miR-138	Increased expression	Knockdown of miR-138 induced apoptosis and prevented tumorigenesis.
Dong (2014) [38]	miR-21	Increased expression	Targeting of PTEN 3′ UTR demonstrated that miR-21 targets the 3′ UTR of pro-apoptotic PTEN, decreasing PTEN mRNA levels.
Chen (2017) [2]	miR-211-5p	Decreased expression	Overexpression of miR-211-5P inhibited proliferation and metastasis by targeting a sequence within the 3′-UTR of SETBP1.
Da Silva (2022) [12]	miR-101 and miR-340	Decreased expression	Overexpression of miR-340 reduced proliferation and migration of TNBC cells.
Moro (2023) [39]	miR-877-5P	Increased expression	miRNA inhibitor targeting miR-877-5p reduced viability and growth of TNBC.
Yu (2020) [40]	miR-3613-3p	Decreased expression	Overexpression of miR-3613-3p inhibited proliferation and migration of TNBC cells, with a G1 cell-cycle arrest through targeting of SMAD2 and EZH2.
Soudan (2020) [41]	Hsp90	Increased expression	The combination of PU-H71 (Hsp90 inhibitor) and DHEA (G6PD inhibitor) suppressed Nrf2 and led to apoptosis.
Caldas-lopes (2009) [42]	Hsp90	Increased expression	Use of Hsp90 inhibitor (PU-H71) suppressed growth and induced killing of TNBC cells.
Oh (2017) [43]	Hsp90	Increased expression	BPD (Hsp90 inhibitor) blocked TNBC cell growth by causing cell cycle arrest at G2/M phase, and induced degradation of several oncogenic proteins, leading to apoptosis.
Fang (2023) [44]	DNAJB4	Decreased expression	DNAJB4 knockdown confirmed the pro-tumorigenic effect by inhibiting TNBC apoptosis and promoting tumor growth. Overexpression of DNAJB4 yielded opposite outcomes.
Muniz-lino (2014) [45]	RhoGDI-2	Increased expression	Targeting RhoGDI-2 with RNA interference resulted in mitochondrial dysfunction, activation of caspase-3 and -9, and increased sensitivity to cisplatin therapy.
Campagna (2023) [46]	PON2	Increased expression	PON2 knockdown led to decrease in cell proliferation.
Wang CQ (2017) [47]	FSCN1	Increased expression	Co-treatment with FSCN1 siRNA and gefitinib resulted in reduced FSCN1 expression compared to individual treatments.
Cox (2019) [11]	PDLIM2	Increased expression (especially in epithelial-to-mesenchymal subtype)	Suppression of PDLIM2 reversed epithelial-to-mesenchymal subtype and inhibited tumor growth.
Giricz (2022) [48]	GRB7	Increased expression	Inhibition of GRB7 resulted in reduction in cell motility and invasion, and promotion of cell death through apoptosis.
Liu (2021) [49]	CLU and PRKD3	Increased expression	Silencing of CLU and PRKD3 suppressed tumor growth.
Ferraro (2013) [50]	EGFR	Increased expression	Downregulation led to reduction in motility, signaling, and proliferation of TNBC cells.
Linklater (2016) [51]	MET and EGFR	Increased expression	Inhibition of MET and EGFR with crizotinib and erlotinib inhibited tumor growth and diminished fluctuations in treatment response compared to individual therapies.
Verma (2017) [52]	EGFR	Increased expression	Targeting of EGFR and nonreceptor tyrosine kinase PYK2/FAK inhibited proliferation of TNBC cells and suppressed tumor growth in a mouse model. PYK2 knockdown induced upregulation of NDRG1, which enhanced NEDD4-HER3 binding, leading to HER3 degradation.
Beatty (2018) [53]	Glutathione	Decreased expression	The use of γ–glutamylcysteine ligase inhibitors to limit glutathione precursors effectively suppressed glutathione levels and inhibited TNBC growth.
Wang (2022) [54]	Glycerophospholipids and unsaturated fatty acids	Dysregulated	Dandelion extract reduced expression of CHKA, resulting in inhibition of PI3K/AKT pathway and its downstream targets SREBP and FADS2.
Pelicano (2014) [55]	Mitochondrial respiration and glycolysis	Decreased mitochondrial respiration and increased glycolysis causing low expression of p70S6K	Reintroduction p70S6K in TNBC cells reversed metabolism to active phosphorylation state.
Lai (2021) [56]	YBX1	Increased expression	Knockdown of YBX1 resulted in suppression of glycolytic genes, reduced expression of EMT-related genes, and decreased tumor migration and invasion.
Apostolidi (2021) [57]	PKM2	Increased expression	Manipulation of phosphorylated PKM2 revealed a connection between the phosphorylation of PKM2 at S37 and the CDK pathway. Treatment with TEPP-46, CDK inhibitor dinaciclib, or their combination, reduced tumor growth and decreased PKM2pS37.
Yang (2011) [58]	FZD7 (Wnt signaling pathway gene)	Upregulation	Knockdown of FZD7 with shRNA led to a reduction in cell proliferation, inhibition of invasiveness and colony formation through silencing of the canonical Wnt signaling pathway.
Christensen (2017) [9]	NF-kB	Upregulation	Inhibition of NF-Kb signaling pathway led to a decrease in a mammosphere formation and tumor initiation.
Dey (2013) [59]	Wnt/ β-catenin genes	Upregulation	Disruption of the Wnt/β catenin pathway through Wnt antagonists showed a direct link between pathway disturbance and metastasis-related characteristics in TNBC.
Malone (2020) [5]	Lipocalin-2 (LCN2)	Increased expression	The use of an LCN2 antibody inhibited TNBC cell growth and migration.
Lobba (2018) [60]	CD90	Increased expression	Overexpression of CD90 in normal mammary cells and knockdown in malignant cells demonstrated that CD90 is involved in morphological change, increased cell proliferation, invasiveness, metastasis, and activation of the EGFR pathway.
Ring (2018) [61]	CBP/β-Catenin/FOXM1	Increased expression	Targeting of CBP/ β-Catenin/FOXM1 with ICG-001 eliminated cancer stem cells and sensitized TNBC tumors to chemotherapy.
Leong (2015) [62]	B7-H4	Increased expression	The use of anti-B7-H4 produced tumor regression in cell line and patient-derived xenograft models of TNBC.
Du (2020) [63]	LAG3 and PD-1	Increased expression	In a mouse model of TNBC, the dual blockade of LAG3 and PD1 led to a better anti-tumor effect rather than either one alone.
Chatterjee (2023) [7]	NARD1, RAD51, PALB2	Increased expression	Amygdalin, a natural glycosidic inhibitor, had a preferential binding for the BRCT domain of the BARD1 receptor.
Shen (2019) [64]	Integrin and EGFR	Increased expression	Tinagl1 inhibited EGFR and integrin/FAK activation and suppressed growth and metastasis.
Cinar (2022) [13]	Serotonin 5-HT7 receptor	Increased expression	Genetic and pharmacological inhibition of 5-HT7 by siRNA and metergoline suppressed TNBC cell proliferation and FOXM1 and its downstream mediators, including eEF2-Kinase and cyclin-D1.
Makvandi (2015) [65]	Sigma-2 receptor	Increased expression	Targeting the sigma-2 receptor with a cytotoxic payload induced caspase 3/7 activation and led to cell death.
Nagano (2014) [66]	Ephrin receptor A10	Expression in TNBC but not in normal tissue	Administration of an anti-EphA10 monoclonal antibody suppressed tumor growth in a xenograft mouse model.
Forte (2018) [67]	CDCP1 (and PDGF-BB/PDGFRβ–mediated pathway)	Increased expression	Knockdown of PDGFRβ in TNBC cells impaired CDCP1 increase induced by WHF treatment, highlighting the role of its receptor as a central player of the WHF-mediated CDCP1 induction.
Ring (2020) [68]	EP300	Increased expression	EP300 knockdown abolished the cancer stem cell phenotype by reducing ABCG2 expression.
Ruiu (2021) [69]	Teneurin 4 (TENM4)	Increased expression	TENM4 silencing impaired tumorsphere-forming ability, migratory capacity, and focal adhesion kinase phosphorylation.
Toosi (2018) [70]	EPHB6	Increased expression	Suppression of either ERK or OCT4 activities blocks EPHB6-induced pro-proliferative responses
Weng (2019) [71]	MCT-1/miR-34a/IL-6/IL-6R signaling axis	Increased expression	MCT-1 knockdown and tocilizumab synergistically inhibited TNBC stemness. Tumor suppressor miR-34a was induced upon MCT-1 knockdown that inhibited IL-6R expression and activated M1 polarization.
De Santis (2022) [8]	BLC6	Increased expression	Inhibition of BCL6 significantly reduced tumor cell growth. Additionally, BCL6 silenced cells were impaired by pharmacological inhibition of the Notch signaling.

## Data Availability

Not applicable.
